# Owner-Related Reasons Matter more than Behavioural Problems—A Study of Why Owners Relinquished Dogs and Cats to a Danish Animal Shelter from 1996 to 2017

**DOI:** 10.3390/ani10061064

**Published:** 2020-06-19

**Authors:** Janne B. H. Jensen, Peter Sandøe, Søren Saxmose Nielsen

**Affiliations:** 1 Department of Veterinary and Animal Sciences, University of Copenhagen, DK-1870 Frederiksberg C, Denmark; zlr736@alumni.ku.dk (J.B.H.J.); pes@sund.ku.dk (P.S.); 2 Department of Food and Resource Economics, University of Copenhagen, DK-1958 Frederiksberg C, Denmark

**Keywords:** animal shelter, canine, cat, Denmark, dog, feline, owner, relinquishment, relinquishment reasons

## Abstract

**Simple Summary:**

Many dogs and cats are relinquished to shelters by their owners. To reduce their number, it will be vital to know whether people relinquish their pets primarily as a result of real or perceived problems created by the animals, or because of problems in their own lives, i.e., whether the reasons are animal or owner related. We therefore investigated reported reasons for giving up cats and dogs to a large shelter in the second-largest city in Denmark, a wealthy Scandinavian country, in the period 1996 to 2017. We observed that owner-related issues were the most common reasons for both cats (74%) and dogs (75%). Problems with the health of the owner were the most common reason, but challenges with housing also contributed significantly, both with cats and dogs. Time pressures were reported by dog owners, but not cat owners. We conclude that the most important way to reduce relinquishments in Denmark, like in other countries studied, will involve focusing on owners—e.g., by finding ways to help them to look after their animals during their own episodes of ill health. Among the animal factors, behavioural problems were by far the most commonly reported reason for relinquishment for both species.

**Abstract:**

Every year, dogs and cats are relinquished to animal shelters by their owners in large numbers. Reasons for relinquishment of dogs and cats to a large Danish shelter from 1996 to 2017 were obtained and characterised. The reasons were available for 86% of the owner-relinquished animals, including 3204 dog relinquishments (90%) and 2755 cat relinquishments (82%). They were allocated to 59 categories, which were further merged into four owner-related and three animal-related reasons. The most commonly reported of these seven reasons for relinquishment of dogs were owner health (29%), animal behavioural problems (23%), housing issues (21%) and lack of time (14%). For cats, the figures were: owner health (32%), housing issues (26%), and animal behavioural problems (25%). No systematic changes in these patterns were found over time. The number of relinquished cats was roughly stable, whereas the number of relinquished dogs decreased on average by 3% per annum. Owner issues were the primary reason for relinquishment in both species, but nearly one-quarter of the animals were relinquished as a result of behavioural problems. As the latter are often connected with the owner in some way, the results emphasise the importance of a focus on owners when addressing pet relinquishment challenges.

## 1. Introduction

Large numbers of privately owned dogs and cats are relinquished to animal shelters annually in Western countries [1,2,3,4,5]. To reduce this traffic and allow more dogs and cats to stay in their homes, there is a need to identify why owners choose to give up their pets [4,5]. In particular, it is important to know whether people relinquish primarily as a result of real or perceived problems caused by the animals or because of problems in their own lives, i.e., whether the reasons are animal or owner related. If behavioural problems and other animal-related problems dominate, the focus should be on giving current and prospective owners realistic expectations about what it requires to live with different kinds of dogs and cats, and teaching them how to prevent, correct, or live with what are perceived as problematic behaviours. If problems with the owners’ life situations prevail, we will need to focus more on helping people to manage their dogs or cats when they have difficulties with, for example, their own health or housing.

An editorial from a veterinary journal concerning unwanted dogs and cats [6] claims that “previous studies have indicated the importance of animal behaviour as the main reason for relinquishment and failed adoptions”. However, whether this is actually true depends on how the numbers are added up. According to a meta-analysis comparing the proportion of dogs surrendered to shelters for dog-related and owner-related reasons, behavioural problems was the most frequently reported reason (in eight out of nine studies), with frequencies of reported behavioural problems as the reason for relinquishment ranging from 11 to 34%. Owner health was reported as a reason in five studies, ranging from 4 to 9%, while moving was a reason reported in four studies, ranging in frequencies from 7 to 48%. Moving and human expectations were reported as reasons in four studies, where these reasons constituted 7 to 48% and 6 to 21% of relinquishments, respectively. Costs were reported as a reason of relinquishment in five studies, with frequencies ranging from 0.6 to 27%. However, since the reporting of the reasons was not systematic in the different studies included, it can be a challenge to compare them overall. In general, the most frequent reasons relate to the owner. Consequently, behavioural problems of the animals were the most consistently reported, but not the most common reasons that owners have given for relinquishing their dog to a shelter. Three of the nine studies were based on shelter records or databases, while the rest were based on questionnaires. Of the studies included in the meta-analysis, five were from the US, two were from the UK, one was from Australia, and one was from Serbia. Overall, even though behaviour problems played an important role for owner relinquishments of dogs, owner-related reasons, according to the meta-analysis, seemed to be more common than animal-related reasons [7].

There are no systematic reviews on why owners choose to relinquish cats to shelters. However, in published studies, the reasons show a similar pattern to that found for dogs, i.e., owner-related factors are more often cited than animal-related factors. Reasons for relinquishment reported from shelters in Sweden, where more than one reason could be given, were that the owner had an allergy to cats (58%); the owner was moving (27%); age, illness or death of owner (24%); new family situation (20%); tired of pet (11%), and lack of time (9%). Abnormal animal behaviour was also mentioned, but no specific statistics were available. A potentially varying definition of abnormal animal behaviour was said to explain why it was hard to quantify across shelters [8]. Accommodation-related (47%), financial (24%) and other personal reasons (28%) were reported as the most common reasons by 55 cat owners relinquishing their cats to an Australian shelter. Animal-related reasons were less common and included unwanted kittens (19%), cats’ behaviour (16%) and cats’ health (8%) [9]. Another study of 2804 owned cats relinquished to an Australian shelter reported that 70% of the relinquishments were due to too many cats in the household. Reasons related to the owners predominated in the remaining 834 cat cases: unable to care for (31%), moving/accommodation issues (27%), and behaviour issues (12%) [10]. Human lifestyle (35%), human housing issues (26%), and animal behaviour problems not including aggression (21%) were the most common reasons for relinquishment of cats to 12 US shelters in the period 1995–1996 [11]. A more recent UK study reported that among 4169 owner relinquishments of cats, 1175 (28%) were due to owner circumstances such as moving (50% of the 1175), owner dying (15%), owner sick (14%), divorce (11%) and financial (7%). Unwanted kittens accounted for 21%, behaviour problems for 11%, and multiple factors under the umbrella “can’t cope” for 7%. The remaining categories included transfer from other facilities (14%), allergy/asthma (7%), pregnancy/young child (3%), too many cats (3%), and cat pregnant or unwell (2%), while no reasons were given for the remaining 5% [12]. From these studies, even more vividly than in the case of dogs, a picture emerges in which the most common reasons why owners choose to relinquish their cats to shelters relate to their own life situation, not to problems relating to the cats.

In this article, we present findings about why owners chose to relinquish their dogs and cats to a large Danish shelter in the period 1996 to 2017. The data presented are based on the records of the shelter and therefore likely to be more accurate than studies based on questionnaires filled out by people working at shelters. They also related to a part of the world, Scandinavia, which, apart from one Swedish cat study, has so far not been studied. Finally, the data set is unique in covering two decades. Other studies cover at most two years.

The objective of the study presented in this paper was to characterise the reasons for which Danish cat and dog owners relinquished their pets and determine whether these were primarily owner or animal related. A secondary aim was to establish whether the reasons for owner relinquishment changed over time.

This study’s principal findings were that the main reason for relinquishment was owner-related for both dogs (75%) and cats (74%), and that poor owner health was the most common reason for relinquishment (29% for dogs and 32% for cats). This picture remained constant over the two decades studied. However, although animal behaviour is not the main cause of relinquishment to shelters in Denmark, it was given as the explanation in approximately one-quarter of the cases.

## 2. Materials and Methods

Based on data from a previous study of 20 organisations managing in total 52 animal shelters based in Denmark [13], a single shelter, which was able to provide access to all records on relinquished dogs and socialised cats, including the reasons for relinquishment, from 1996 to 2017, was identified. The shelter manages both dogs and cats without specialising in either. It is located in the second largest city (Aarhus) in Denmark, and has a catchment covering both metropolitan and rural areas. The shelter receives animals handed in by owners or others with relation to the animal, stray animals, and animals in temporary custody of the police. It charges fees for taking owned dogs and cats that currently vary from USD 35 to 125, depending on neuter and health status. There are no public shelters in Denmark. The shelter we focused on is owned by a large Danish non-governmental organisation and is considered a limited admissions shelter.

All the relinquishment records were manually inspected by J.B.H.J. and categorised by reason for relinquishment. This study included data on two types of relinquishment: reason for first-time relinquishment of an animal and reason for relinquishment of an animal previously adopted from the shelter. All of the animals, except 6 dogs and 13 cats (which were relinquished by the police), were relinquished by their owners or owners’ next of kin and were considered owner relinquished.

Forty-seven reasons for relinquishment were specified prior to the onset of the data analysis, and 12 reasons were added during assessment. The selection of these 59 reasons was based on examination of the existing literature [11,14,15] and seven years of personal experience working at the shelter [J.B.H.J.]. The 59 reasons were condensed into four owner-related factors: owner health (including specific physical health issues such as asthma and non-specific reasons such as owner sick or old), housing issues (including moving to work abroad or travelling), lack of time, and lack of interest (including home breeding with subsequent relinquishment of surplus puppies and kittens from backyard breeding); and three animal-related factors, including behaviour problem, physical state (health and physical characteristics), and other. These reasons are listed detailed in the Results section. Where the accurate reason for and date of relinquishment were not given, the reason was categorised as unknown. To describe the temporal trend in each of the seven main factors, the proportions of each reason of relinquishment for each species were plotted over time.

Initial data compilation was followed up by several visits to the shelter, and any irregularities, such as missing information or irregular dates, were checked directly in the shelter’s filing system. Records of animals indicating stays in the shelter of more than 450 days were excluded (n = 31) to avoid including duplicated records, as this length of stay was considered improbable by the shelter staff (except for one dog, which was kept for 489 days under police instructions).

The proportion of reasons for relinquishment was estimated for each stratum (dog, dog owner, cat, and cat owner), excluding animals where no reason had been specified. The overall reasons for dog vs. dog owner and cat vs. cat owner were compared using the z-score test for comparison of two proportions. Linear trends over time were assessed using logistic regression, where relinquishment was used as outcome, and the year of relinquishment as an explanatory factor in a model for each of the seven main factors for cats and dogs, separately. We used the glm()-function in R (R Foundation for Statistical Computing, Vienna, Austria). A *p* <0.05 was considered statistically significant, and Bonferroni correction was used to correct for the multiple comparisons performed.

## 3. Results

### 3.1. Overall Numbers and Reasons for Relinquishment of Dogs and Cats

**Figure 1:**
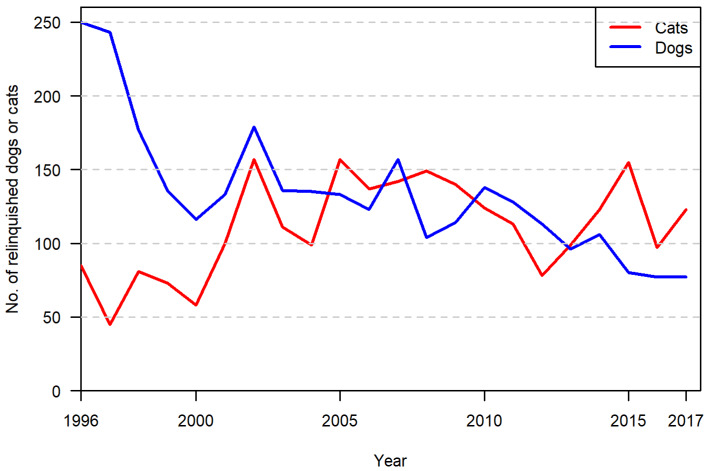
Annual number of owner-relinquished dogs and cats at a large Danish shelter from 1996 to 2017.

**Figure 2:**
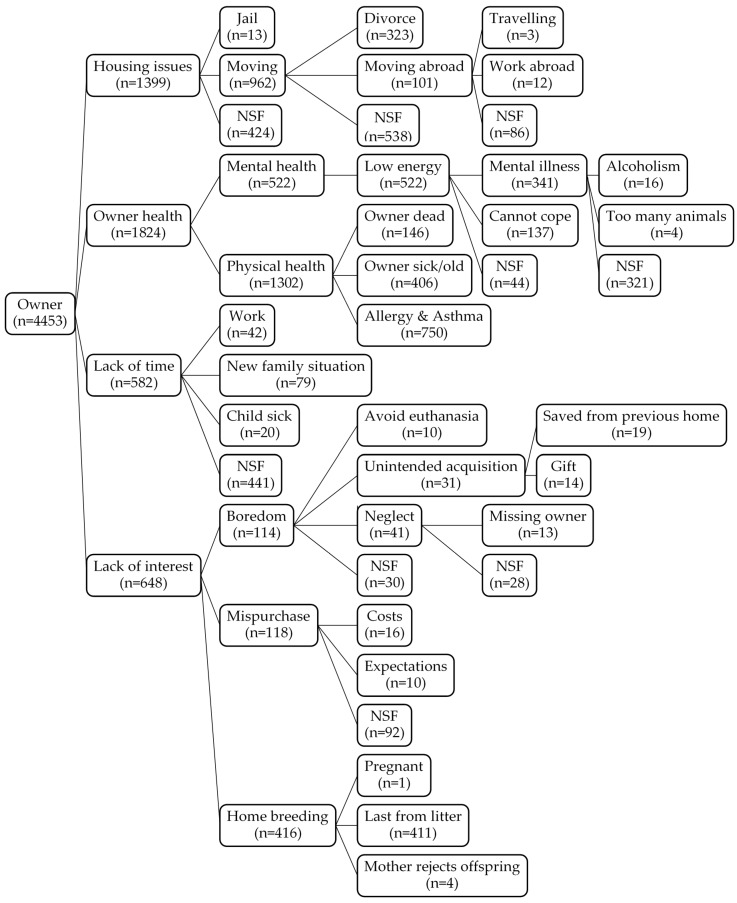
Reasons for relinquishment related to the owner from 1996 to 2017. Illustration of the merging and characterisation of owner-related reasons for relinquishment of dogs and cats. NSF: not specified further.

**Figure 3:**
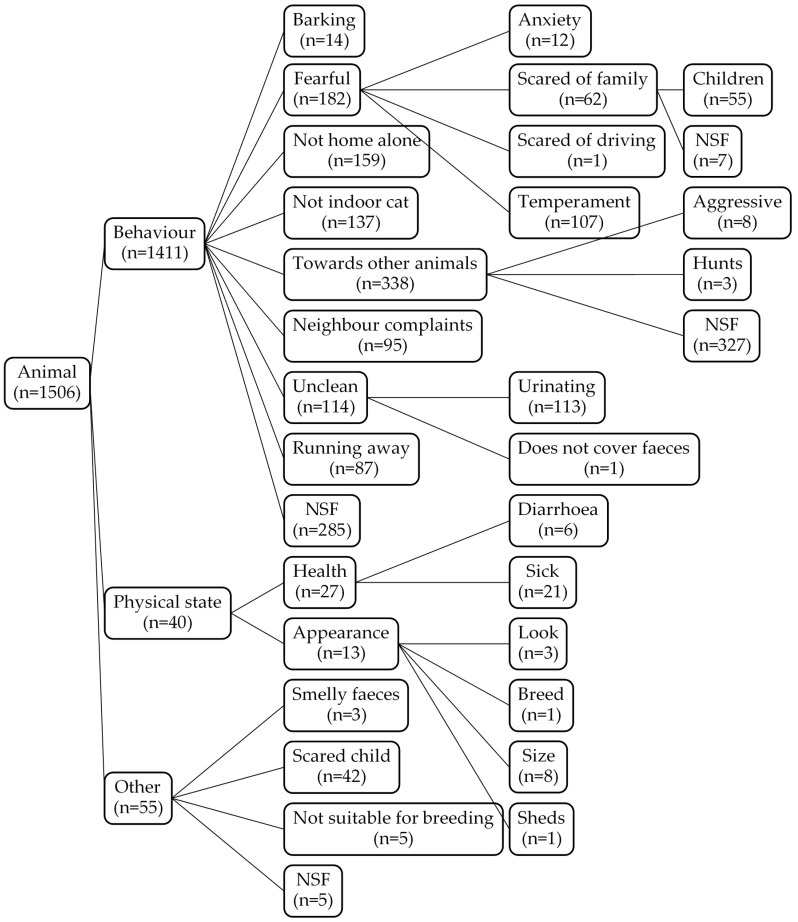
Reasons for relinquishment related to the animal from 1996 to 2017. Illustration of the merging and characterisation of animal-related reasons for relinquishment. NSF: not specified further.

Data (Supplementary Materials) on reasons for relinquishment were available for 3204 of a total of 3559 cases of owner relinquishment of dogs and litters (90%) and 2755 of a total of 3374 cases of owner relinquishment of cats and litters (82%). In total, 5959 explanations of relinquishment were collected—of which, 4613 referred to first-time relinquishments and 1346 referred to the return of a previously adopted animal to the shelter. Annual numbers of owner-relinquished dogs and cats are given in Figure 1. Clear recorded reasons were unavailable for 897 relinquishments, and 77 included no dates. These records are ignored in the figures. Owner-related reasons for relinquishing cats and dogs are depicted in Figure 2, with the corresponding animal-related reasons listed in Figure 3. A total of 32 owner-related reasons and 27 animal-related reasons are included. Some of these were relatively detailed; others were of a more general nature without further specification. Furthermore, some specific reasons (e.g., rehabilitation) were not included as a specific category, because they were already included in another category (e.g., rehabilitation occurred due to substance abuse following mental illness).

### 3.2. Relinquishment of Dogs

**Figure 4:**
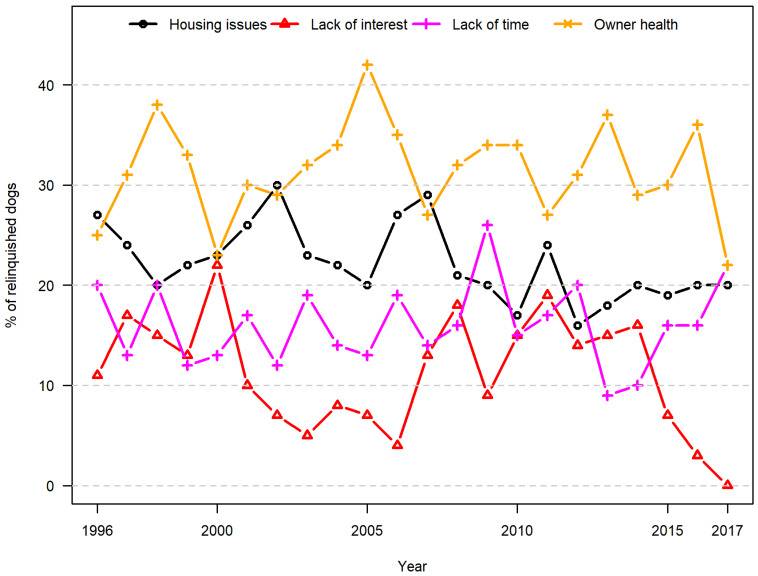
Owner-related reasons for relinquishment (dogs). Proportional distribution of reported owner-related reasons for the relinquishment of dogs in a Danish animal shelter from 1996 to 2017.

In the period under investigation, the most common owner-related reasons for relinquishment were poor health of the owner (29%), housing issues (21%), and lack of time for the dog (14%) (Table 1). The proportion of dogs relinquished due to owner health ranged from 22% in 2000 and 2017 to 42% in 2005, while the proportion of dogs relinquished due to housing issues did not change overall (Figure 4). 

**Figure 5:**
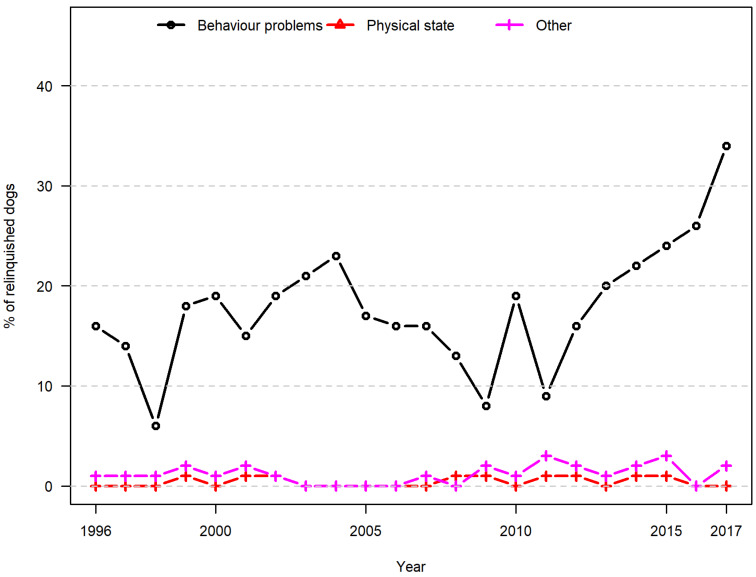
Animal-related reasons for relinquishment (dogs). Proportional distribution of reported animal-related reasons for the relinquishment of dogs in a Danish animal shelter from 1996 to 2017.

In dogs, behaviour problems were the most frequently reported animal-related reason for relinquishment (23%), varying between 6% in 1998 and 34% in 2017. Less than 1% of dogs were relinquished due to the physical state of the animal (Figure 5). The observed variations over time did not appear to be systematic (Figure 4 and Figure 5), and assessment of the time trends revealed no linear increase or decrease over time for any of the factors.

### 3.3. Relinquishment of Cats

**Figure 6:**
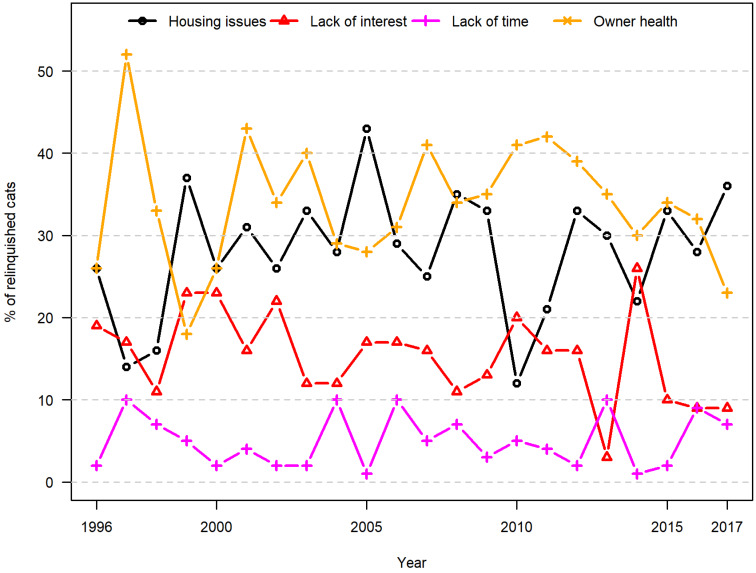
Owner-related reasons for relinquishment (cats). Proportional distribution of reported owner-related reasons for the relinquishment of cats in a Danish animal shelter from 1996 to 2017.

**Figure 7:**
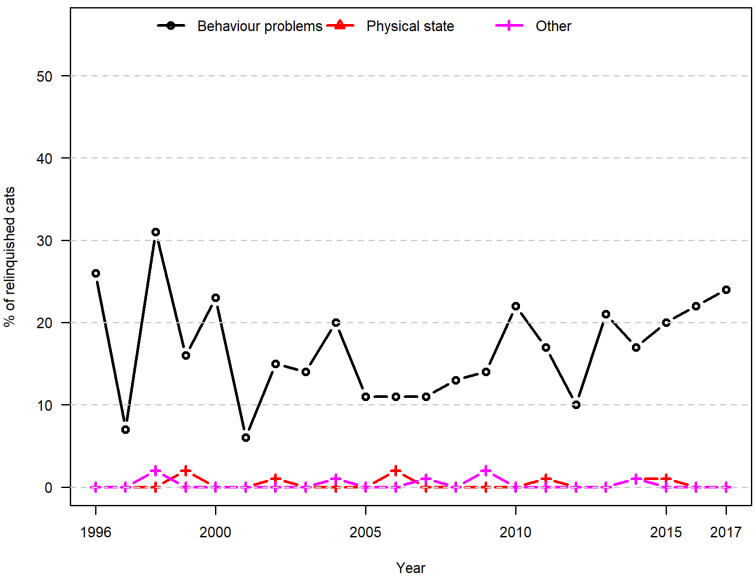
Animal-related reasons for relinquishment (cats). Proportional distribution of reported animal-related reasons for the relinquishment of cats in a Danish animal shelter from 1996 to 2017.

From 1996 to 2017, the most commonly reported reasons for relinquishing cats were poor health of the owner (32%) and housing issues (26%), followed by behavioural problems (25%) (Table 1). Variation over time appeared to be random (Figure 6). An exception was the factor lack of time, which the logistic regression model suggested had decreased overall by 22% over the 22 years. However, obvious fluctuations in the patterns observed in Figure 6 suggested that this pattern should be considered to be random. In all years other than 1996, cats were more likely to be relinquished as a result of owner health and housing issues than for reasons of lack of time and lack of interest (Figure 6). Cats were relinquished more often as a result of behaviour problems than they were for other animal-related reasons in the investigated period (Figure 7). The absolute numbers, however, ranged from 2 to 26 each year; and with such low numbers, it is no surprise that trends over time appear as random.

### 3.4. Owner and Animal Factors Compared

Overall, 75% of cats were reported to have been relinquished for owner-related reasons and 25% for animal-related reasons. The corresponding figures for dogs were 74% and 26%, respectively. The differences between the owner- and animal-related factors (Table 1) were all statistically different (*p* < 0.0001), except for returned animals, where no difference was observed. The primary reason for returning an animal previously adopted from the shelter was behaviour problems (48%) (Table 1).

## 4. Discussion

### 4.1. Discussion of Findings

The primary finding of this study is that the reasons owners gave for relinquishing their dogs or cats to shelters most often referred to their own situation (notably health and housing issues). Reasons referring to the animals were given less often. Behaviour problems were nevertheless given as reason in many cases, and at 23%, they were the second most frequently given reason after owner health (29%) in the case of dogs. In the case of cat relinquishment, the reasons for relinquishment were (in descending order) owner health (32%), housing issues (26%) and behaviour problems (25%).

More dogs (14%) than cats (4%) were relinquished due to lack of time. This may be because dogs require daily walks, need to be let out at regular intervals, and (possibly) because they need more social stimulation than cats do. In Denmark, cats often roam freely [16] and/or have access to a cat flap. This study also looked at developments and change over more than twenty years. During this period, no systematic variation in the reasons given for owner relinquishment was identified. Although there were fluctuations over time, the number of cats relinquished by their owners at the shelter studied seems to have been reasonably stable over the period, whereas the number of dogs decreased. This is in line with findings from a previous study of 90% of Danish shelter capacity covering the period 2004–2017 [13]. The recent historical pattern of owner relinquishments to the shelter covered in the present study therefore seems to be in line with that observed in other Danish shelters. The observed decrease in owner relinquishment of dogs, but not cats, to shelters in the last part of the period studied can be explained in terms of the private rehoming and selling of dogs via social media and websites that have become available in that period. The reason why this new opportunity has been used in the case of dogs but not so much in the case of cats is probably that prices for dogs generally are much higher than those for cats, and that there is a surplus of unwanted cats in Denmark. The higher prices for dogs are linked to a robustly regulated pet sector in Denmark with a ban on the selling of dogs from shops and strict requirements on large breeding facilities [17]. Therefore, there is a greater demand for dogs offered for sale privately on social media and websites than there is for cats [13]. 

The predominance of housing as an issue is probably also linked to the mixture of rental apartments with and without permission to keep pets found in Denmark [18]. Usually, local regulations state whether or not pets are allowed. However, in some countries, there is a move towards allowing pets in homes for the elderly, as these may have a beneficial effect on such residents [19]. Of course, this may lead to the relinquishment of companion animals when the owner passes away. This has yet to be studied. Housing issues are highlighted in most of the literature we examined (see Introduction). 

Still, animal-related problems account for nearly one-quarter of the owners’ relinquishments in our study. A difficulty with our distinction between owner- and animal-related reasons is that behavioural problems can be human-related in origin: that is, they may be caused by poor ownership, limited understanding of the animal’s needs and bad care and management. An animal left alone indoors for long periods of time may have no other choice than inappropriate elimination or destructive behaviour for enrichment. In Denmark, 17% of cats are kept indoors at all times [16], and a Danish study has found an increased risk of behavioural problems in indoor cats [20]. Similarly, it has been found that 36% of cat owners keep more than one cat [16], although fighting among cats living together is not uncommon, which in turn may be perceived as a behavioural problem. Studies have suggested that behavioural problems in pets are associated with intact status [21,22], but an estimated 86% of Danish privately owned cats are neutered [16]. At present, we lack data on the neuter status of Danish dogs.

There is an interesting contrast between our findings of why owners choose to relinquish their companion animals to a shelter and findings from three Danish studies [23,24,25] from three subsequent decades, beginning in the 1990s, of why owners choose to have their dogs euthanized by a vet. While in our study, owner-related reasons were more common, these studies all found that behaviour problems, notably aggression, were three to four times more common than reasons related to the owner’s situation. So, it seems that Danish dog owners are more likely to relinquish a dog to a shelter when there is a problem relating to their own situation and more likely to have the dog euthanized when the dog’s behaviour is the issue.

A special concern, also covered by our study, relates to returning animals previously adopted from the shelter, where behaviour problems is the most common reason given for owner relinquishment, accounting for 48%. This emphasises the need for shelters to carefully assess the suitability of the match between the new owner and the animal prior to adoption.

Previous studies see the lowering of owner expectations and the provision of education for new owners as the solution to surplus pets ending up in shelters [3,6]. We agree that focused educational efforts might raise awareness among owners of the real drivers of certain animal behaviours. Better understanding, more realistic expectations and timely interventions might salvage a human–animal bond before it reaches breaking point.

### 4.2. Limitations of This Study

Lambert et al. [7] list primary sources of study bias, including the source of the study population, the methods of measurement, and author-reported bias. As regards the first of these, the population in this study was based on data from one large Danish animal shelter covering both rural and metropolitan areas with high- and low-income households. In Denmark, there are several kinds of shelters, differing in size, adoption policies and management. However, the amount of data we gathered and the long study period can be assumed to provide a good baseline from which general conclusions about the reasons for pet relinquishment in Denmark can be drawn. Thus, the shelter we studied was considered representative of Danish shelters more generally given its location and size, and over the 22 year span of this study, very little variation occurred, which supports the general representativeness of the results, although this cannot be assessed further at this point. Of 6933 relinquishments, 974 (14%) lacked either a reason or a date for the relinquishment. This might have skewed the results, affecting our measurement of the primary reasons for relinquishment, but it would not have had a serious impact on conclusions about whether it was the animal or the human that was the primary factor in the relinquishments, and, to this extent, it did not have a significant impact on the results.

With methods of measurement, several challenges need to be acknowledged. First, a specific reason for relinquishment may be understood differently by the relinquisher and the shelter staff, and this can lead to different definitions. This has also been identified as an issue previously [26]. The inclusion of only one shelter and our focus on reasons over time reduced this problem, but it remains a difficulty where comparisons with other shelters are concerned. On the other hand, most of the concepts involved in our study are relatively straightforward. 

More importantly, there is rarely just one reason for relinquishment, and this may have caused some skewness in the results. For example, the reason reported may be moving house, but perhaps no effort was made by the owner to find a pet-friendly new house because the animal had also been difficult to house train. Thus, it has been argued that the reasons companion animals are relinquished are typically multifactorial [27]: first, the demographics are important. First-time owners are more likely to relinquish than others. Second, the way the pet is acquired plays a role, with “unintentional owners” being more likely to relinquish their pets than those that gave thought to the acquisition. The characteristics of the pet (age, size, and breed) and behavioural problems can also be a factor when a pet is relinquished. Lastly, personal circumstances, or problems the owner has, are listed as a category, and it is suggested that these may be more dynamic than the other categories because external factors such as loss of a job, income or housing may affect the decision to relinquish the pet.

Because just one reason was reported in most cases, the possibility of several reasons playing a role could not be considered via our findings. In a study such as ours, this may not be methodologically desirable, as it was only the reasons that the staff deemed more important or dominant that were available to be considered. In addition, there is a risk of social desirability (SD) bias [28]. An owner may, for example, report allergy to be the main reason for relinquishment, rather than admitting that he or she was unable to handle the animal-related problem. Moreover, owners may believe that shelters are unwilling to accept especially difficult pets. Staff at the shelter we investigated confirmed this possibility. Quite often they expressed caution, or outright scepticism, about the reasons for relinquishment provided by owners. Moreover, it was indeed true that the shelter did not accept every single animal brought in. A number of dogs and cats deemed unsuitable for rehoming were refused admittance. However, the number involved seems to have been very small and was not considered significant.

Other literature on the relinquishment of pets looks at characteristics of relinquished animals. As this study directly investigates the reasons reported during relinquishment, it is one way in which this study is expected to give more accurate results. However, in other studies investigating owner-reported reasons, the researchers spoke directly to the relinquishers, while our results rely solely on the reports of shelter staff with no scientific background. These reports were not originally gathered for research purposes, moreover, and thus important information may have been lost, and further elaboration was unavailable. It is worth noting that interviewing relinquishers confidentially after relinquishment may help to mitigate SD bias, as the answers are more likely to be straightforwardly honest: the interviewee will at this point have little or no reason to misrepresent the situation.

This study was longitudinal in nature and temporal changes should be identifiable if present. We used logistic regression to identify linear effects of time, but none could be identified. We concluded that there was no temporal trend, but we also note that the fluctuations were marked with a small proportion followed by a high followed by a low in most cases. These fluctuations were mostly caused by relatively few observations within each category at specific time points. Therefore, logistic regression is not very likely to capture these. Time series analyses could also have been used, but visual assessment was deemed more appropriate given the small number of observations at each time point.

More research is needed on this topic. Ideally, it will include in-depth interviews with relinquishers, as these will help to identify possible combinations of relinquishment reasons, demographic trends among owners, and possibly behavioural evaluations of the relinquished animals.

## 5. Conclusions

From 1996 to 2017, we found that dogs were most often relinquished as a result of (in order of importance) owner health, behaviour problems and housing issues, while cats were most often relinquished following owner health, housing issues and behaviour problems. Both dogs and cats were relinquished mainly for reasons connected with the owner. To prevent some of these relinquishments in the future, it will be necessary to find ways to help people keep their pets despite housing issues, poor health and other problems that they face in their lives. As behaviour problems are still a common reason for relinquishing both dogs and cats, more education and awareness raising for owners, and the lowering of owner expectations, could resolve a part of the problem. Our findings may hopefully help to target future interventions tackling the issue of surplus pets in Denmark and similar countries.

## Figures and Tables

**Table 1:** Comparison of owner- and animal-related reasons for relinquishment. Overall stratified distribution of owner- and animal-related reasons for relinquishment of dogs and cats to a Danish animal shelter, and the distribution of reasons for first-time relinquishment (first-time rel.) and for returning an animal previously adopted from the shelter. Unknown reasons were not included in the calculations. The *p*-value expresses the probability that the observed proportion of owner-related factors was equal to the observed proportion of animal factors.

Main Factors	Dogs		Cats		Overall		First-Time rel.		Returned	
	*n*	*%*	*n*	*%*	*n*	*%*	*n*	*%*	*n*	*%*
**Owner related**	2414	75	2039	74	4453	75	3793	82	660	49
Owner health	938	29	886	32	1824	31	1489	32	335	25
Housing issues	679	21	720	26	1399	23	1179	26	220	16
Lack of time	464	14	118	4	582	10	521	11	61	5
Lack of interest	333	10	315	11	648	11	189	4	43	3
**Animal related**	790	25	716	26	1506	25	820	18	686	51
Behaviour problem	732	23	679	25	1411	24	767	17	644	48
Physical state	17	0.5	23	0.8	40	0.7	18	0.4	18	2
Other	41	0.8	14	0.5	55	0.9	35	0.8	20	2
Z-score/*p*-value	25.2/<0.0001		22.7/<0.0001		34.6/<0.0001		36.4/<0.0001		−0.73/<0.0001	

## Supplementary Materials

The following are available online at https://www.mdpi.com/2076-2615/10/6/1064/s1, Table S1: Raw data.
